# Antibiotic use among twelve Canadian First Nations communities: a retrospective chart review of skin and soft tissue infections

**DOI:** 10.1186/s12879-020-4842-1

**Published:** 2020-02-10

**Authors:** Dahn Jeong, Ha Nhan Thi Nguyen, Mark Tyndall, Yoko S. Schreiber

**Affiliations:** 1 School of Epidemiology, Public Health and Preventative Medicine, University of Ottawa, Ottawa, Ontario Canada; 2 Ottawa Hospital Research Institute, Ottawa, Ontario Canada; 3 First Nations and Inuit Health Branch, Ontario Region, Health Canada, Ottawa, Ontario Canada; 4 BC Centre for Disease Control, Vancouver, British Columbia Canada; 5 School of Population and Public Health, University of British Columbia, Vancouver, British Columbia Canada; 6 Department of Medicine, The Ottawa Hospital and University of Ottawa, Ottawa, Ontario Canada; 7 Sioux Lookout Meno Ya Win Health Centre, 1 Meno Ya Win Way, PO Box 909, Sioux Lookout, ON P8T 1B4 Canada

**Keywords:** First nations, Community-acquired MRSA, Rural health, Skin and soft tissue infection, Antimicrobial use

## Abstract

**Background:**

Previous publications indicated an emerging issue with community-acquired methicillin-resistant *Staphylococcus aureus* (CA-MRSA), particularly skin and soft tissue infections (SSTIs), in Indigenous communities in Canada. The objectives of this analysis were to explore the prevalence of SSTIs due to CA-MRSA and patterns of antimicrobial use in the community setting.

**Methods:**

A retrospective chart review was conducted as part of an environmental scan to assess antibiotic prescriptions in 12 First Nations communities across five provinces in Canada including Alberta, Saskatchewan, Manitoba, Ontario, and Québec. Charts were randomly selected from nursing stations and patients who had accessed care in the previous 12 months and were ≥ 18 years were included in the review. Data was collected from September to December, 2013 on antibiotic prescriptions, including SSTIs, clinical symptoms, diagnostic information including presence of CA-MRSA infection, and treatment.

**Results:**

A total of 372 charts were reviewed, 60 from Alberta, 70 from Saskatchewan, 120 from Manitoba, 100 from Ontario, and 22 from Québec. Among 372 patients, 224 (60.2%) patients had at least one antibiotic prescription in the previous 12 months and 569 prescriptions were written in total. The prevalence of SSTIs was estimated at 36.8% (137 cases of SSTIs in 372 charts reviewed). In 137 cases of SSTIs, 34 (24.8%) were purulent infections, and 55 (40.2%) were due to CA-MRSA.

**Conclusions:**

This study has identified a high prevalence of antibiotic use and SSTIs due to CA-MRSA in remote and isolated Indigenous communities across Canada. This population is currently hard to reach and under-represented in standard surveillance system and randomized retrospective chart reviews can offer complimentary methodology for monitoring disease burden, treatment and prevention.

## Background

The health of First Nations populations is a priority for the Canadian government, and for First Nations themselves [1]. Disproportionately high rates of communicable and non-communicable diseases have been documented within Canadian First Nations communities [2–6]. The disease burden is exacerbated by environmental determinants of health (e.g. food insecurity, water safety, congested and unstable housing, unemployment), individual determinants of health (e.g. smoking, alcohol use disorder, drug use, diabetes), social determinants of health including a history of social and psychological trauma of colonialism [7, 8], and limited or discriminatory access to health care resources [5, 9, 10].

In Canada, the Federal Action Plan on Antimicrobial Resistance (AMR) and Use was launched to monitor AMR, determine the full magnitude of the problem, and to evaluate the appropriate use of antibiotics [11]. The incidence of Methicillin-Resistant *Staphylococcus aureus* (MRSA) has been rising, with disproportionate rates of community-associated (CA) MRSA documented among First Nations populations living in remote or rural areas, in Canada [12]. Muileboom et al. [13] reports in their five-year review of laboratory and patient data in northwestern Ontario that in 2012, 56% of *S. aureus* isolates were CA-MRSA. They also found that in 2011, the rate of CA-MRSA infection was 2482 per 100,000 people [13]. Furthermore, based on the Canadian Nosocomial Infection Surveillance Program (CNISP) data, the rate of MRSA colonization and incidence increased from 2.12 per 1000 admitted patients in 2013 to 2.35 per 1000 in 2017. They also note a trend in increase of CA-MRSA and a decrease of hospital-acquired (HA) MRSA [14]. The appropriate diagnosis and treatment of skin and soft-tissue infections (SSTIs) are critical for curtailing infections caused by CA-MRSA, and this is particularly important among First Nations, who often live in remote and isolated areas with limited access to health services with high burden of SSTIs.

Capturing timely and accurate information from remote and isolated First Nations communities remains an ongoing challenge for infectious disease surveillance systems. As a result, more information is needed on prevalence of CA-MRSA infections, SSTIs, including information on clinical presentation, diagnoses and treatments. The objectives of this analysis were to: 1) Identify the prevalence and patterns of SSTIs, including those caused by CA-MRSA, and antibiotic prescriptions; 2) Estimate the prevalence, clinical presentation and diagnostic information of SSTIs, and 3) Assess the appropriateness of antibiotic prescriptions for SSTI cases.

## Methods

### Study design and sample selection

This study was a retrospective chart review conducted at nursing stations in First Nations communities in five Canadian provinces (Alberta, Saskatchewan, Manitoba, Ontario, Québec). As part of a quality improvement project at Health Canada, communities were invited to participate on a voluntary basis and were enrolled in consultation with the Regional Nursing Officers (RNO). At the request of the communities, the facility names and communities are not identified to ensure confidentiality. A simple random sample of patient paper charts was selected by choosing a random starting point in the charts drawer and pulling out the charts to be reviewed in an evenly-spaced manner until the required number of charts to be review was reached. The review was conducted by research team (DJ, HN, YS) independent of the nursing stations. Patients charts were included in the review if they had accessed care in the previous 12 months and were ≥ 18 years of age. Any time an antibiotic was prescribed during this one-year period, it was captured in the Antibiotic Tracking Case Report Form (CRF) for analysis. Patient names and unique identifiers were not recorded on the antibiotic tracking data sheet, as each antibiotic tracking data sheet was assigned a number. There was no personal information or personal identifiers in the data collection. At the time of the study design, there were a total of 85 nursing stations with the First Nations and Inuit Health Branch (FNIHB) at Health Canada. The sample size calculation was based on 12 nursing stations (14.1%) being able to participate in the study and calculated the estimated proportion of antibiotic use. $\sqrt{N}$-proportional allocation strategy produced numbers of charts that were needed in each province to capture the minimum number of cases (i.e. infections treated with antibiotic(s)): 45 in AB, 55 in MB, 30 in ON, 20 in BC, 35 in QC and 35 in SK). The number of charts needed was calculated based on unpublished internal audit conducted by Health Canada in 2011 on antibiotic use in the First Nations communities, it was conservatively estimated that 25% of patient charts included antibiotic prescriptions for treatment. In order to estimate the proportion of correct antibiotic treatment within a margin of error of 10%, the minimum number of antibiotic cases required to sample was calculated as *n* = 96 rounded to 100. Based on the estimate from the internal audit, we assumed 1 in 4 charts would contain an antibiotic case, hence the number of charts required was calculated as *n* = 400 to find 100 charts containing an antibiotic case. Charts were randomly selected at a rate of approximately 20 per 1000 population in the nursing station catchment area. Upon reviewing 372 charts, we found 224 charts that documented at least one antibiotic case in the previous 12 months, we ceased collecting additional data.

### Measures

SSTIs were measured as both a patient-based estimate (e.g. the number of patients with at least one SSTI in the previous 12 months) and case-based estimate (e.g. the total number of unique SSTI cases in the previous 12 months, to account for repeat infections).

Demographic variables included gender and age. Clinical variables included pre-existing conditions such as diabetes, cardiac disease, alcohol use disorder, renal disease, pregnancy, liver disease, Chronic Obstructive Pulmonary Disease (COPD), immune-compromise, and/or asplenia, and presence of a medical device (e.g. urinary catheter, central line, or other).

Information on etiologic agent was collected for each unique SSTI case. CA-MRSA was defined based on the standardized epidemiological case definition proposed by US Centers for Disease Control and Prevention (CDC) [15], and the diagnosis of CA-MRSA was either confirmed by wound culture associated with the SSTI episode or presumed by existing MRSA colonization, based on previous wound culture results or an indication of MRSA colonization in the patient’s chart. Other etiologies included Methicillin-Sensitive *Staphylococcus aureus* (MSSA), other organism, or unknown.

Primary SSTI symptoms were categorized as purulent (abscesses, boils, folliculitis, impetigo, open wounds, sores with pus or purulent discharge), non-purulent (cellulitis, any SSTI with swelling or redness but no pus or discharge), skin breakdown (lacerations, rashes, scabs, warts and abrasions), wounds (open sores, ulcers, bite wounds and surgical site infections), or unknown. All cases were considered SSTI in the present analysis as they were treated as infections with antibiotic(s) prescription at the nursing stations. Treatment options included wound care, incision and drainage, prescribed antibiotic, type of antibiotic (topical only, topical and oral, oral only, and intravenous), antimicrobial combination therapy, and adjunctive therapy. Clinical outcomes were categorized as cure, failure, improvement and unknown. Data was extracted on diagnostic tests including wound culture, blood culture, complete blood count (CBC), no diagnostic test, and missing.

An evaluation of appropriateness of antibiotic use for SSTI was conducted by subject experts (YS, HN) using two guidelines - the Clinical Practice Guidelines (CPG) administered by the FNIHB [16] and the Infectious Diseases Society of America (IDSA) guidelines [17]. Evaluations were based on the patients’ pre-existing medical conditions, clinical symptoms, and availability of diagnostic test results including culture and susceptibility as applicable. In cases where multiple antibiotics were used in combination, the appropriateness for the combination therapy was determined. Appropriateness was assessed based on choice of antibiotic (e.g. use of narrow or broad-spectrum agent), dosing of antibiotic (e.g. higher or lower dose than indicated), duration of prescription (e.g. longer or shorter duration) and use of combination therapy. Overall appropriateness was assessed based on the appropriateness of choice, dosing, duration of antibiotics, use of combination therapy, as well as the patient’s predisposing medical conditions.

### Statistical analyses

Patient-based analyses calculated the prevalence of antibiotic use by dividing the number of patients with any antibiotic prescription by the total patient-charts sampled. Case-based analyses used the total number of unique SSTIs to summarize etiologic agents, diagnostic testing, clinical symptoms, outcomes, and appropriateness of the prescribed antibiotics. All data were analyzed using SAS statistical software, version 9.3 [18].

## Results

A total of 372 patient-charts from 12 nursing stations were randomly selected and reviewed, 60 from Alberta, 70 from Saskatchewan, 120 from Manitoba, 100 from Ontario, and 22 from Québec (Table 1). There were 224 (60.21%) patient-charts with at least one antibiotic prescription in the previous 12 months. Among the 224 patients, there were 569 individual antibiotic prescriptions in total. Of the 224 patients, there were 86 patients (38.39%) with at least one prescription of antibiotic(s) for treatment of SSTIs in the past 12 months. Among the 86 patients with at least one SSTI, there were 137 individual SSTI episodes in the past 12 months: 59 patients had one episode of SSTI, 18 patients had two SSTI episodes, three patients had three SSTI episodes, three patients had four SSTI episodes, two patients had five SSTI episodes, and one patient had 11 episodes of SSTI. There were 27 (31.40%) patients who had more than one episode of SSTI recorded in the past 12 months.

**Table 1:** Geographic distribution of patient-charts and individual SSTI cases

Province	Total patient charts reviewed	Total number of patient-charts that contained at least one prescription of antibiotic(s)	Number of patient-charts that contained at least one SSTI case^a^	Total number of cases recorded from patient-charts	Number of SSTI cases recorded from patient-charts	Total number of antibiotic prescriptions	Prevalence of antibiotic use
Alberta	60	39	7	77	8	89	0.650
Saskatchewan	70	40	16	63	21	81	0.571
Manitoba	120	64	29	170	60	207	0.533
Ontario	100	68	24	126	34	162	0.680
Quebec	22	13	10	23	14	25	0.591
Total	372	224	86	459	137	569	0.602

^a^Case was defined as a charted visit at the nursing station where an antibiotic prescription of one or more types of antibiotics was provided in the past 12 months

Table 2 presents the patient-based analysis and the characteristics of the 224 patients with any antibiotic prescription in the past 12 months. There were 133 (59.38%) female patients and 95 (42.41%) were between 18 and 30 years. The most common pre-existing conditions were diabetes (21.43%), cardiac disease (18.75%) and alcohol use disorder (17.86%). When comparing patients with at least one antibiotic prescription for SSTIs (*n* = 86) vs. all 224 patients, the groups were characteristically similar, except the prevalence of pregnancy, as there were no pregnant women in the SSTI patient populations (*p* = 0.0416).

**Table 2:** Characteristics of patient-charts with an antibiotic prescription in the last 12 months (*n* = 224)

Variable	All patients with at least one antibiotic prescription		Patients with at least one antibiotic prescription for SSTI		*p*-value
	Total *N* = 224		Total *N* = 86		
	n	(%)	n	(%)	
Gender					0.0939
Male	91	(40.63)	44	(51.16)	
Female	133	(59.38)	42	(48.84)	
Age					0.6956
18–30	95	(42.41)	39	(45.35)	
31–40	51	(22.77)	15	(17.44)	
41–50	40	(17.86)	18	(20.93)	
51–60	20	(8.93)	10	(11.63)	
61–70	12	(5.36)	2	(2.33)	
70+	6	(2.68)	2	(2.33)	
Creatinine Clearnance					0.8741
< 60 mL/min	10	(4.46)	5	(5.81)	
> 60 mL/min	197	(87.95)	75	(87.21)	
Missing	17	(7.59)	6	(6.98)	
Pre-existing conditions					
Diabetes	48	(21.43)	18	(20.93)	0.9235
Cardiac disease	42	(18.75)	19	(22.09)	0.5074
Alcohol use disorder	40	(17.86)	20	(23.26)	0.2814
Renal disease	13	(5.80)	7	(8.14)	0.4535
Pregnancy	12	(5.36)	0	(0.00)	0.0416
Liver disease	10	(4.46)	4	(4.65)	1.000
COPD	6	(2.68)	2	(2.33)	1.000
Immuno-compromised	3	(1.34)	2	(2.33)	0.6198
Asplenia	0	(0.00)	0	(0.00)	–
Other disease	103	(45.98)	38	(44.19)	0.7762
Presence of medical device					
Urinary catheter	1	(0.45)	1	(1.16)	0.4785
Central line	0	(0.00)	0	(0.00)	–
Other device	5	(2.23)	2	(2.33)	1.0000

Table 3 presents the case-based analysis on the 137 unique SSTI cases. In the 55 MRSA positive cases, 27 were confirmed by wound culture during the recorded SSTI episode and 28 were presumed by previous colonization with MRSA as indicated with a history of MRSA infection in the patient charts. Other organisms isolated from wound cultures included Group A *streptococcus* (*n* = 9), *Viridans* group *streptococcus* (*n* = 1), *Enterobacter cloacae* (*n* = 2), and *Serratia marcescens* (*n* = 1). In 69 cases of SSTI, the organism was unknown as culture was not applicable or test results were missing. Among the 137 SSTIs, 34 (24.82%) were purulent, 15 (10.95%) were non-purulent, 34 (24.82%) were classified as wounds and 37 (27.01%) infections remained unknown. Incision and drainage was performed on 12 (8.76%) SSTIs: on eight purulent infections (23.53% of all purulent infections, *n* = 8/34), and on one non-purulent infection (6.67% of all non-purulent infections, *n* = 1/15). Oral antibiotics were the most commonly prescribed (62.77%), then topical only (17.52%) followed by IV (8.76%) and both topical and oral (8.76%).

**Table 3:** Clinical characteristics of each unique SSTI case (*n* = 137)

Variables	n	%
Etiologic agent		
MRSA^a^	55	(40.15)
MSSA	5	(3.65)
Other organism	8	(5.84)
Unknown organism	69	(50.36)
Primary SSTI symptom		
Purulent^b^	34	(24.82)
Non-purulent^c^	15	(10.95)
Skin breakdown^d^	17	(12.41)
Wounds^e^	34	(24.82)
Unknown	37	(27.01)
Wound care		
Yes	67	(48.91)
No	70	(51.09)
Incision & drainage		
Yes	12	(8.76)
Purulent	8	(5.84)
Non-purulent	1	(0.73)
Skin breakdown	0	(0.00)
Wounds	0	(0.00)
Unknown	3	(2.19)
No	125	(91.24)
Type of antibiotic		
Topical only	24	(17.52)
Topical and oral	12	(8.76)
Oral only	86	(62.77)
IV	12	(8.76)
Missing	2	(1.46)
Combination therapy		
Yes	41	(29.93)
No	96	(70.07)
Adjunctive therapy		
Yes	68	(49.64)
No	69	(50.36)
Clinical Outcomes		
Cure	16	(11.68)
Failure	19	(13.87)
Improvement	17	(12.41)
Unknown	84	(61.31)

^a^27 MRSA cases confirmed by wound culture, 28 MRSA presumed by colonization, as indicated by a history of MRSA infection in the patient chart

^b^Purulent SSTI included: 10 abscesses, 1 cyst (with drain), 1 folliculitis, 11 boils, 1 impetigo (probable) and 10 other infections with purulent discharge

^c^Non-purulent SSTI included: 10 cellulitis, 1 mastitis and 4 other infections with swelling/redness but no discharge

^d^Skin breakdowns included: 8 lacerations, 2 scabs, 2 abrasions, 2 warts, 2 rashes, 1 infected skin graft

^e^Wounds included: 27 wound infections with no discharge and 7 bite wounds

Table 4 summarizes the diagnostic testing characteristics. Only 46 out of 137 (33.58%) underwent any diagnostic testing. The majority (61.31%) of SSTI cases did not receive any diagnostic testing. The antibiotic susceptibility of MRSA, shown in Table 5, was assessed from wound culture and susceptibility results when they were available in the patient charts (*n* = 21). Only 29% of MRSA isolates were susceptible to erythromycin, but most were susceptible to clindamycin and co-trimoxazole (90.5 and 95.2%), which also suggests that most MRSA infections were probably due to CA-MRSA phenotypically, in addition to the epidemiological case definition [15].

**Table 4:** Diagnostic testing characteristics for each unique SSTI case (*n* = 137)

Diagnostic test	n	(%)
Blood culture	2	(1.46)
Complete Blood Count (CBC)	4	(2.92)
Wound culture (*n* = 40)^a^	40	(29.20)
Purulent	9	(22.50)
Non-purulent	3	(7.50)
Skin breakdown	1	(2.50)
Wounds	9	(22.50)
Unknown	18	(45.00)
No diagnostic test	84	(61.31)
Missing	7	(5.11)

^a^Sub-division of infection characteristic by would culture sub-sample (*n* = 40). Definitions of sub-categories were: purulent infections – abscesses, boils, folliculitis, impetigo and open wounds or sores with pus or purulent discharge. Non-purulent infections – cellulitis and other infections with swelling or redness but no pus or discharge. Skin breakdowns – lacerations, rashes, scabs, warts and abrasions. Wounds – open sores, ulcers, bite wounds and surgical site infections

**Table 5:** Antibiotic susceptibility (%) of MRSA (*N* = 21)

Antibiotic susceptibility	Erythromycin	Clindamycin	Tetracycline	Co-trimoxazole	Vancomycin	Oxacillin	Gentamicin	Linezolid	Cefazolin	Penicillin
Number of MRSA isolates (%)	6 (28.6)	19 (90.5)	21 (100)	20 (95.2)	21 (100)	0	21 (100)	21 (100)	0	0

Table 6 contains the assessment of the overall appropriate use of antibiotics to treat each individual SSTI. Overall appropriateness was assessed for MSSA/MRSA infections and by prescriber types. Inappropriate therapy resulting in ineffective treatment was more common in SSTIs due to MSSA or MRSA.

**Table 6:** Overall appropriateness of treatment for SSTI cases

Overall appropriateness	Inappropriate but not resulting in ineffective therapy, n (%)	Appropriate, n (%)	Inappropriate resulting in ineffective therapy, n (%)	Unable to assess, n (%)
SSTIs (*n* = 134^a^)	20 (14.93)	82 (61.19)	20 (14.93)	12 (8.96)
MRSA (*n* = 55)	11 (20.0)	28 (50.91)	14 (25.45)	2 (3.64)
MSSA (*n* = 5)	0 (0)	1 (20.0)	1 (20.0)	3 (60.0)
By prescriber type (*n* = 131^a^)				
RN	11 (11.96)	58 (63.04)	16 (17.39)	6 (6.52)
NP	0 (0)	8 (80.00)	0 (0)	2 (10.00)
MD	9 (29.03)	16 (51.61)	4 (12.90)	1 (3.23)

^a^Missing data was excluded from analysis

## Discussion

Delivering health care to remote and isolated Indigenous communities continues to be a public health priority in Canada. Almost 37% of our patients were seeking care for SSTIs at nursing stations. Most patients were presenting with purulent or open wounds. Of critical concern, CA-MRSA was documented in over 40% of SSTIs, either by wound culture or presumed by previous colonization. Although research around skin infections in Indigenous communities is limited, this high burden of infection aligns with the current literature; in Australia, researchers have found that 75% of residents from two remote Australian Indigenous communities visited a primary healthcare centre with a skin infection at least once in a year, and skin infections were the dominant reason for visiting a primary healthcare centres in Indigenous communities [19]. They also found MRSA rates around 50% in the north (of Australia) [20], and highlighted the high burden of skin infections in Indigenous communities in Australia. The findings of this study adds to the literature and emphasize the need to improve health equity for First Nations populations and support for nursing stations to providing prompt and adequate care to meet population needs.

This study has identified a high annual prevalence of antibiotic use in nursing stations with over 60% of patients having at least one antibiotic prescription over the previous 12 months. In Canada, it was reported that 660 to 683 antibiotic prescriptions per 1000 inhabitants were dispensed in the communities between 2010 and 2012 [21]. In this review, there were almost 600 antibiotic prescriptions written to 224 patients, indicating a high level of repeat infections. More detailed documentation and laboratory investigation are needed to identify if re-infection is attributed to failure of therapy, persistent carriage or re-infection. While our study cannot speak directly to the larger determinants of health faced by our patient population, the level of reinfections emphasizes the need to improve infection prevention and control practices that go beyond medical treatment administered at the nursing stations. Ongoing health needs that influence infection rates include housing conditions, access to clean water, and availability of adjunctive services [22].

Appropriateness of antibiotic prescription was evaluated in terms of choice of agent, dose, frequency and during using Health Canada CPG and the IDSA clinical guidelines. While there was a high degree of agreement between CPG and IDSA with regards to choice of antibiotic, there was considerable discordance with respect to dosing and duration. This should be taken into consideration for providers and surveillance systems when conducting comparative analyses. In addition, higher incidence of purulent SSTIs (e.g. abscesses), highlight the importance of antibiotic sparing treatment approaches, such as incision and drainage.

In Canada, the proportion of CA-MRSA strains have been steadily increasing [23]. Investigations of MRSA trends in Canada found that 23% of MRSA infections identified in hospitals were determined to be community-associated, based on the CA-MRSA case definition by CDC or based on the absence of healthcare-associated risk factors [23]. In our community setting, MRSA was documented in 40.15% of SSTIs seen at nursing stations, and 31.40% of SSTIs were re-infections. This finding is similar to a study among First Nations in Ontario that found that 56% of *Staphylococcus aureus* infections were CA-MRSA strains, and 25% were re-infections [13]. While hospital-based surveillance of MRSA is able to identify CA-MRSA infections, the discrepancy in estimates sourced from hospitals versus community clinics highlights the value of using multiple population-level surveillance methods that can routinely capture information from populations that are highly affected by CA-MRSA. Additionally, the CNISP only includes large metropolitan hospitals, and community hospitals–while smaller clinics go unevaluated; a surveillance system at community-level would enable the monitoring of antibiotic susceptibility patterns in the communities that may differ from those seen in hospital settings. In larger centres, infection prevention and control initiatives typically focus on monitoring hospital-acquired infection. Using prescription database is problematic, as antimicrobials are often not dispensed by prescription through a pharmacy, but provided on-site from stock at the nursing stations. While purchasing data may provide an indication of how much of a certain antibiotic was requested, one cannot infer that it was used for treatment as opposed to be removed from stock due to limited shelf-life. Many antibiotic treatments are provided by nursing staff without consultation with a physician (as per FNIHB CPG), therefore billing data would not capture these antibiotic prescription events. We aimed to contribute a different perspective to the existing literature by gathering data directly from the communities, which are usually excluded from such types of studies. Although the sample size is not large, we tried to portray a representative picture of the epidemiology of CA-MRSA SSTIs in the Indigenous communities by sampling data from 12 First Nations communities across five provinces.

Participation in this study was voluntary and it is possible that communities with higher SSTIs or CA-MRSA prevalence would be more or less likely to participate, limiting the generalizability of our findings. Antibiotic utilization patterns were defined by prescription only and we are unable to comment on uptake or adherence, introducing measurement bias. The chart review only includes patients who accessed and received care, potentially excluding patients who are most vulnerable. Additionally, the blinding of reviewers was not possible as we had to review paper charts in the nursing station. However, the authors had no previous interaction with patients and staff in the visited nursing stations and have no conflict of interest. As this was a retrospective analysis, the availability of data was limited: inconsistent availability of microbiology reports, lack of consistent description of the clinical presentation, lack of documentation of follow-up (lack of information on patient outcomes), missing information about patient’s allergies (missing or not updated) and current prescriptions (e.g. for chronic diseases). This large proportion of missing information may have biased the results. For example, missing information on the etiologic agent (about 40% of SSTI cases were confirmed as MRSA infections and the etiologic agent remained unknown in 50% of the cases) might underestimate the prevalence of MRSA in SSTIs and the true proportion might be higher than estimated in this study. Prevalence of MRSA has been documented as high as 50% in some regions [24]. Patient charts also lacked important social and environmental risk factors for SSTIs including overcrowding, housing and access to clean running water. Finally, due to logistical challenges, the ratio of sampled charts did not completely meet the designed sampling ratio; however, the minimum required number of cases was met in all provinces included in this analysis.

## Conclusions

This study is filling an important data gap on SSTIs, CA-MRSA, and antibiotic use in First Nations communities in Canada. We have observed a high annual prevalence of antibiotic use among inhabitants living in remote and isolated communities seeking treatments in nursing stations. Many of these community members are seeking care for SSTIs, with over 40% of cases attributable to CA-MRSA infection. In addition to appropriate and timely medical treatment, a comprehensive approach to infection control and prevention is needed to ensure that First Nations living in remote and isolated communities achieve equitable healthcare services.

## Data Availability

The datasets generated and analysed during the current study are not publicly available due to privacy and confidentiality of individuals and communities included in the study but are available from the corresponding author on reasonable request.

## Abbreviations

AMR: Antimicrobial Resistance (AMR)
CA-MRSA: Community-associated (CA) MRSA
CBC: Complete blood count
CNISP: Canadian Nosocomial Infection Surveillance Program
COPD: Chronic Obstructive Pulmonary Disease
CPG: Clinical Practice Guidelines
CRF: Case Report Form
FNIHB: First Nations and Inuit Health Branch
HA-MRSA: Hospital-acquired (HA) MRSA
IDSA: Infectious Diseases Society of America
MRSA: Methicillin-Resistant *Staphylococcus aureus* (MRSA)
MSSA: Methicillin-Sensitive *Staphylococcus aureus*
OHSN-REB: Ottawa Health Science Network Research Ethics Board
REB: Research and Ethics Board
RNO: Regional Nursing Officers
SSTI: Skin and soft-tissue infections

## References

[1] Health Canada. A Statistical Profile on the Health of First Nations in Canada: Determinants of Health, 2006 To 2010. Ottawa; 2014; p. 1-59. Available from: https://www.canada.ca/en/indigenous-services-canada/services/first-nations-inuit-health/reports-publications/aboriginal-health-research/statistical-profile-health-first-nations-canada-determinants-health-2006-2010-health-canada-2014.html.

[2] Gordon J, Kirlew M, Saginur R, Bocking N, Kelly L, Kennedy C (2015). Fever in our first nations. Can Med Assoc J.

[3] Gordon J, Kirlew M, Schrieber Y, Saginur R, Bocking N, Blakelock B (2015). Acute rheumatic fever in First Nations communities in northwestern Ontario: Social determinants of health “bite the heart.”. Can Fam Physician.

[4] Mashru J, Kirlew M, Saginur R, Schreiber YS (2017). Management of infectious diseases in remote northwestern Ontario with telemedicine videoconference consultations. J Telemed Telecare.

[5] He H, Xiao L, Torrie JE, Auger N, Gros-Louis N, Zoungrana H (2017). Disparities in infant hospitalizations in indigenous and non-indigenous populations in Quebec. Canada Can Med Assoc J.

[6] Jongbloed K, Pearce ME, Pooyak S, Zamar D, Thomas V, Demerais L (2017). The cedar project: mortality among young indigenous people who use drugs in British Columbia. Can Med Assoc J.

[7] Lavoie JG (2013). Policy silences: why Canada needs a National First Nations, Inuit and Metis health policy. Int J Circumpolar Health.

[8] McNally M, Martin D (2017). First nations, Inuit and Métis health: considerations for Canadian health leaders in the wake of the truth and reconciliation Commission of Canada Report. Healthc Manag Forum.

[9] Siddiqi A, Shahidi FV, Ramraj C, Williams DR (2017). Associations between race, discrimination and risk for chronic disease in a population-based sample from Canada. Soc Sci Med.

[10] Jacklin KM, Henderson RI, Green ME, Walker LM, Calam B, Crowshoe LJ (2017). Health care experiences of indigenous people living with type 2 diabetes in Canada. Can Med Assoc J.

[11] Public Health Agency of Canada. Federal Action Plan on Antimicrobial Resistance and Use in Canada: Building on the Federal Framework for Action. Ottawa; 2015; p. 1-18. Available from: https://www.canada.ca/content/dam/canada/health-canada/migration/healthy-canadians/alt/pdf/publications/drugs-products-medicaments-produits/antibiotic-resistance-antibiotique/action-plan-daction-eng.pdf.

[12] Loewen K, Schreiber YS, Kirlew M, Bocking N, Kelly L (2017). Community-associated methicillin-resistant Staphylococcus Aureus infection: literature review and clinical update. Can Fam Physician.

[13] Muileboom J, Hamilton M, Parent K, Makahnouk D, Kirlew M, Saginur R (2013). Community-associated methicillin-resistant Staphylococcus aureus in Northwest Ontario: a five-year report of incidence and antibiotic resistance. Can J Infect dis med Microbiol [Internet].

[14] Public Health Agency of Canada (2015). Antimicrobial Resistant Organisms (ARO) Surveillance. Summary Report for Data from January 1, 2009 to December 31, 2014 [Internet].

[15] Morrison MA, Hageman JC, Klevens RM (2006). Case definition for community-associated methicillin-resistant Staphylococcus aureus [2]. J Hosp Infect [Internet].

[16] First Nations and Inuit Health Branch, Health Canada. Clinical Practice Guidelines for Nurses in Primary Care. Ottawa: Government of Canada; 2012. Available from: https://www.canada.ca/en/indigenous-services-canada/services/first-nations-inuit-health/health-care-services/nursing/clinical-practice-guidelines-nurses-primary-care/adult-care.html.

[17] IDSA SDL, Bisno AL, Chambers HF, Dellinger P, Goldstein EJ (2014). Practice guidelines for the diagnosis and Management of Skin and Soft Tissue Infections: 2014 update by the infectious disease society of America. Clin Infect Dis.

[18] SAS Institute Inc. SAS Statistical Software version 9.3 [Computer software]. Institute Inc. Cary; 2017.

[19] Thomas L, Bowen AC, Ly M, Connors C, Andrews R, Tong SYC (2019). Burden of skin disease in two remote primary healthcare centres in northern and Central Australia. Intern Med J.

[20] Bowen AC, Daveson K, Anderson L, Tong SY. An urgent need for antimicrobial stewardship in Indigenous rural and remote primary health care. Med J Aust [Internet]. 2019; Available from: https://onlinelibrary.wiley.com/doi/abs/10.5694/mja2.50216; mja2.50216. 10.5694/mja2.50216 31155725

[21] Public Health Agency of Canada. Canadian Antimicrobial Resistance Surveillance System 2017 Report [Internet]. Ottawa; 2018. Available from: https://www.canada.ca/content/dam/phac-aspc/documents/services/publications/drugs-health-products/canadian-antimicrobial-resistance-surveillance-system-2017-report-executive-summary/CARSS-Report-2017-En.pdf

[22] Tosas Auguet O, Betley JR, Stabler RA, Patel A, Ioannou A, Marbach H (2016). Evidence for community transmission of community-associated but not health-care-associated methicillin-resistant Staphylococcus Aureus strains linked to social and material deprivation: spatial analysis of cross-sectional data. PLoS Med.

[23] Simor AE, Gilbert NL, Gravel D, Mulvey MR, Bryce E, Loeb M (2010). Methicillin-resistant Staphylococcus aureus colonization or infection in Canada: National Surveillance and changing epidemiology, 1995-2007. Infect Control Hosp Epidemiol.

[24] Schreiber Y, Jeong D, Matsumoto C-L, Gordon J, Farrell T, Bocking N. Burden of *S. aureus* Infection in Northwestern Ontario: 2008–2015 (ABSTRACT). Off J Assoc Med Microbiol Infect Dis Canada [Internet]. 2018;3S Available from: http://jammi.utpjournals.press/doi/pdf/10.3138/jammi.3.suppl.01.fm.

